# What Was the Cause of the Death of Geo. A. Gardner?

**Published:** 1880-01

**Authors:** Wm. A. Pease

**Affiliations:** Dayton, Ohio


### WHAT WAS THE CAUSE OF THE DEATH OF GEO. A. GARDNER?

I noticed the statement of the death of Mr. Geo. A. Gardner, of Brooklyn, N. Y., at the time the statement appeared in the papers, the comments of interviewers and the interviewed upon it, some of whom were professors in medical colleges, and they were so unsatisfactory and incomplete; so lacking in thoroughness and professional comity, that I awaited the appearance of the Dental Journals with some interest, hoping that their notice of the unfortunate occurrence might be so broad and comprehensive as to leave little more to be desired. They are now before me, and I still feel that something more remains to be said; that they have not sufficiently analyzed the subject; shown the incompleteness of the diagnosis; if diagnosis there were, the inadequacy of the cause to the effect, and more than all, the want of professional courtesy that could saddle such a charge as that upon a kindred profession, on so poorly defined grounds. They have not vindicated the professional standing as they should, so that medical men shall pause and weigh well their words, before they cast the imputation of carelessness, to say nothing of incompetency upon a member of their profession; but this can hardly be expected from a profession, a considerable proportion of whose members are not respectful to one another, but, who bandy epithets backwards and forwards between themselves, like a shuttle-cock, and when summoned before juries as experts, by their want of a clear conception of their subject, are the tools and the amusement of the lawyers, the muddlers of the jury and the despair of the judge. Charity may step in here in regard to the statement of Dr. Guy, and perhaps it ought, to some extent, and give him the benefit of the excuse of the boy about to be punished, that "he did not go to do it," and very probably he did not, to the full extent. It was, perhaps, an after thought—a convenient excuse to get himself well out of a case that had surprised him by terminating much more seriously than he expected. With a certain class of physicians, post-mortem inventions and subterfuges have to be employed to account for unlooked-for results. But his hands should be very clean, and his record very clear, who would incriminate another and cast a blight upon the practice, if not upon the lives of two hitherto irreproachable practitioners, and lead a distinguished family to believe that it has lost a member by inadvertance or a questionable use of remedies by others than himself. This subject assumes greater and graver proportions the more it is looked at. A man is brought suddenly into notoriety, who seems to be bewildered by it—thrown off from his balance—and in his dazed effort to render himself less unenviably conspicuous, he seeks to throw it upon others. Nay, more, he would, or he has caused, a doubt in the public mind as to the competency of a numerous and honorable profession, his own peers in general and professional education and ability in the treatment of a certain disease, with the appropriate remedy. That disease, toothache, is not seldom. It is general, common, and it has to be treated by hundreds of practitioners for thousands of patients every day, and *arsenic* is the only efficient, practical and almost painless remedy for it. The use of it is venerable with age, sanctioned by high and almost universal authority—a great public want, and it is destined to go down through the ages as a boon to the human race—the best pain-killer for the most intolerable suffering pulpitis, humanity is heir to; and it is safe, it has no history of deaths from its use, as have opium, chloroform and many other remedies that are prescribed by physicians, qualified and unqualified, every day, and that humanity must have. It may be abused, it may be used unnecessarily; science may finally prove that for a certain class of cases, it is not indicated, but it can never be supplanted in the numerous cases where it is indicated, and dentists must be the persons to use it, and they must see to it that no unnecessary odium is attached to their use of it. It is not a heroic remedy in any sense in which it it is used in dental practice, and there have, hitherto, been found no idiosyncrasies that contra-indicate it. If a certain class of cases of exposed pulp can be treated, caped, and the nerve survive, as it seems now probable in a moderate degree, it eliminates that class from the need of arsenic. But why are we discussing the use of arsenic in this case? There is no proof that it was used; nothing but the assertion of a man who has got himself into trouble, unenviable notority, and like a drowning man is catching at a straw. On the contrary, there is the emphatic denial of both of the dentists who treated the patient before Dr. Guy saw him, that arsenic was used, and they are both competent and credible witnesses, certainly more competent than Dr. Guy in the absence of a *post mortem* and the actual presence of the drug in the system. Conjecture will not do, and unsupported assertion is in bad taste, unavailable for a man who has a strong personal reason for using it, when it is contradicted by men who have not the same weighty reasons for denial, supported as they are by the history of the case, so far as we have it, and the concurrent, general practice of dentists. Prof. Taft has sufficiently discussed the improbability of arsenical poisoning, the impossibility of so small an amount as is used by dentists, to produce so disasterous results the want of evidence that Dr. Guy treated for arsenical poisoning, and the omission to state in the certificate of death, that it was due to it.

Dr. Guy; says: "I did not see Mr. Gardner until the 15th of September, four days after he was attacked. The poison had then been actually absorbed." Dr. Waters, of Boston, says: "That he did not use arsenic in Mr. Gardner's tooth, but simply a solution of carbolic acid." Dr. Marvin, of Brooklyn, says:

"I found the tooth temporarily filled; removed the temporary filling, made an application of creosote, hoping thereby to give him relief from pain until he could return to his dentist, which I recommended him to do." This was their treatment, and to every dental mind in the world, it would show that arsenic was not used. Dr. W. further says, *he removed a gold filling, a capping having been placed over the nerve when it was filled, and proceeded to treat for alveolar abscess.* Can anything be clearer than that? Arsenic is used to destroy nerves, not to save them. The dentist who filled the tooth with gold, was laboriously and consientiously trying to save the nerve, and avoid the use of arsenic. He was carrying out the latest theory, that the nerves can be capped and saved, and this is one to the many exceptions to that theory. Whether he did it *secundem artem* or not, according to the latest theories of the nerve-cappers is unknown. Whether he rightly estimated the amount and location of the exposure, the age and temperament that will bear it, [he certainly tried according to the best lights and abilities that he had, and as another evidence of his conscientiousness, he filled the tooth with gold]; whether he malleted it in and thus killed the nerve by concussion, is unknown; but we have one case recorded of death occuring primarily from nerve-capping, and by the blundering treatment of a physician, afterwards. Whether the pulp would have died, if a soft filling had been used, even amalgam, is problematical, but the chances of its survival would have been more numerous.

Let us study the case and make it a text for some observations on similar ones, strengthened by close observation in every day practice and an accumulating experience. The case is very ciear, nothing more could be desired. I refer to it now, when Dr. Waters took charge of it, removed the gold filling and found a discomposing pulp. It is said he applied *traction* to it—what that is, is a mystery. If it means that he partially luxated the tooth, and then left it to remain, created a wound difficult to heal, in addition to the irritation and possible inflammation that was commencing at the end of the roots, there was abundant cause for serious trouble. It is a theory of practice that could hardly have emanated from Harvard University, that was looking down upon him with its calm and venerable eyes. But, he says he did apply "a solution of carbolic acid." Let us see what the reason was for that.

In filling the tooth an effort had been made to save the life of the pulp by capping it, inflammation had supervened and the plug had been removed. The first indication was, to open freely the pulp chamber and remove the partially decomposing pulp, for I assume that the pulp could not have been wholly decomposed, and the immediate result would have been, to remove the pressure on the periosteum at the end of the root, give it rest, and without any other treatment than the placing of a pledget of cotton loosely in the cavity, to prevent the accumulation of food, the periostial irritation would have subsided. Whether he did that or not, is uncertain. Dr. Marvin says: "I found it temporarily filled, removed the temporary filling, made an application of creosote, etc. That temporary filling was the worst thing that could have been done under the circumstances, if it was a tight one, as it probably was, even assuming that he had thoroughly removed the pulp, for the anticedent pressure of gas upon the periosteum might have been sufficient and long continued, to have created a local sub-inflammation, and there might have been a septic element in his blood, seeking a place to focalize, sufficient to have caused a continuance of the inflammation, and the exudation of fluid into the pulp canal to putrefy and generate a gas that could not escape. The temporary filling was placed there in expectancy—it was a sign of doubt, and from all that can be gathered from Dr. Marvin's statement and treatment, the tooth was not in a very bad condition, much less threatening, when he saw it. But valuable time had been lost; the irritation had been too long continued, and the information is not before me as to whether he placed a tight capping over the creosote. What the use of the creosote was, and what curative properties it was supposed to have, is one of the unsolved problems in this case. If it be assumed that at that time there was effusion, and ulceration is more improbable, the difficulty is increased. Nothing can be plainer than that the effusion should be allowed to escape, to relieve tension, and we can not suppose that there was a double track through the pulp canal, the one for the egress of the effusion and the other for the ingress of the creosote. The thing to be done, was to get rid of the effusion, and it ought to have been aided by all the means in his power. The disease must have far progressed, and created a sack of considerable dimensions, to give it force sufficient to drive or elevate the effusion through the canal and overcome the gravity of the creosote, aided by the cap above it. What was wanted was, to keep the effusion in the most liquid form, and remove all impediments to its escape. The effect of creosote would be to coagulate it and make it more difficult to escape, and, if the canal was small, the coagulum might entirely block it up. And if, by any means, as the weight and pressure of the superincumbent cap, the creosote were driven through the foramen, it would destroy the periosteum or the sack, and create such a lesion that after the lapse of four days, when Dr. Guy first saw, it might well make him think that gangrene was present, but it would not frighten a dentist, who is familiar with such conditions, and he would probably have summarily dealt with it, by extracting the tooth; when the cause of the inflammation being removed, the depletion from the bleeding, which might have been increased by the injection of warm water, would have caused the rapid subsidance of the inflammation, and the case would not have obtained public notoriety, and a life would have been saved.

The mistake of Dr. Guy consisted clearly in taking a case that did not belong to him, that he did not understand, and that he was not prepared to treat. Had he relegated it to a dentist, he would have escaped an unenviable notoriety.

Looking at the case in all of its bearings; taking into consideration, all of the facts that the history of it affords, I am compelled to say, that were I sitting as a coroner upon it, my verdict must necessarily be of death from a meddlesome assumption of treating a disease that did not lie within his sphere of practice; that he did not understand, and was not prepared to manage.

WM. A. PEASE,
Dayton, Ohio.

